# Duplication of the left vertebral artery in a patient with dissection of the right internal carotid artery and Ehlers–Danlos syndrome: case report and review of the literature

**DOI:** 10.1007/s12565-012-0152-z

**Published:** 2012-09-07

**Authors:** Michał Polguj, Kazimierz Jędrzejewski, Mirosław Topol, Julia Wieczorek-Pastusiak, Agata Majos

**Affiliations:** 1 Department of Angiology, Medical University of Łódź, Narutowicza 60, 90-136 Lodz, Poland; 2 Department of Normal and Clinical Anatomy, Medical University of Łódź, Narutowicza 60, 90-136 Lodz, Poland; 3 Radiology Department, Medical University of Łódź, Kopcińskiego 22, 90-153 Lodz, Poland

**Keywords:** Computer tomography angiography, Doppler ultrasonography, Duplication, Ehlers–Danlos syndrome, Vertebral artery variation

## Abstract

Duplication of the left vertebral artery was observed in a 43-year-old Caucasian male with dissection of the right internal carotid artery during multidetector 64-row computer tomography and Doppler ultrasonography B-flow mode. Both duplicated segments arose from the left subclavian artery and united at levels C5–C6 to form a single vessel. The presented case describes precisely the origin and diameter of both vertebral arteries. Additionally, after all procedures associated with diagnosis and treatment of the patient, Ehlers–Danlos syndrome type IV was diagnosed. The lumen of the duplicated vertebral artery was smaller than normal; it can be concluded that this variant has clinical implications and should be taken into consideration when vertebral arteries need catheterization.

## Introduction

Variations of the origin and course of the vertebral arteries are uncommon, but extremely important to recognize in the diagnosis, catheter-based evaluation and treatment of patients suffering cerebrovascular disease. One of the rarest anomalies is the duplication of the extracranial segments of vertebral arteries, which is usually an incidental finding in an autopsy series, angiographic studies or, more recently, MR and CT angiography and color Doppler studies (Harnier et al. 2008; Ionete and Omojola 2006; Kendi and Brace 2009; Mahmutyazicioglu et al. 1998).

The term “duplication of the vertebral artery” is applied to a vessel that has two origins with a variable level of fusion in the neck (Goddard et al. 2001; Ionete and Omojola 2006). Nevertheless, the precise definition of this anomaly is occasionally confused with “fenestration”. However, “fenestration of the vertebral artery” refers to an artery with a single origin, with two parallel segments anywhere along its course and fusion (Goddard et al. 2001; Ionete and Omojola 2006). Both “duplication” and “fenestration” are variants of developmental abnormalities resulting from a failure of fetal vessels to involute (Lie 1972; Padget 1948; Sim et al. 2001). The main difference between duplications and fenestrations is that, in duplication, a vertebral artery has two origins, a variable course and fusion level in the neck. In contrast, fenestration refers to a vessel with a single origin, where the main trunk divides into two parallel segments anywhere along its course (Goddard et al. 2001; Harnier et al. 2008).

The frequency of fenestration of the vertebral artery is identified in 0.23–1.95 % of angiographies or autopsy studies (Goddard et al. 2001; Sim et al. 2001). Duplication is much rarer, reported by Bergman et al. (1988) in 0.72 % of studied cadavers.

This anomaly is often associated with significant cerebrovascular abnormalities such as symptomatic kinking, aneurysm, arterio-venous fistulae and arterio-venous malformations (Dare et al. 1997; Gaskill et al. 1996; Kendi and Brace 2009; Koenigsberg et al. 2003; Lie 1972; Thomas et al. 2008). It has been speculated that duplicated vertebral arteries may be more predisposed to dissection (Dare et al. 1997; Gaskill et al. 1996). Therefore, every description of a duplication of the vertebral artery associated with another anomaly or genetic disease is important, as each contributes to a database allowing a meta-analysis to be performed in the future.

## Case report

A 43-year-old Caucasian man was admitted to the emergency department of our hospital for headache associated with weakness of the left limbs. He had no medical history, he denied even slight trauma of the neck, and his only cardiovascular risk factor was tobacco smoking for 10 years. Neurological examination revealed verbal aphasia and left hemiparesis.

**Fig. 1a, b:**
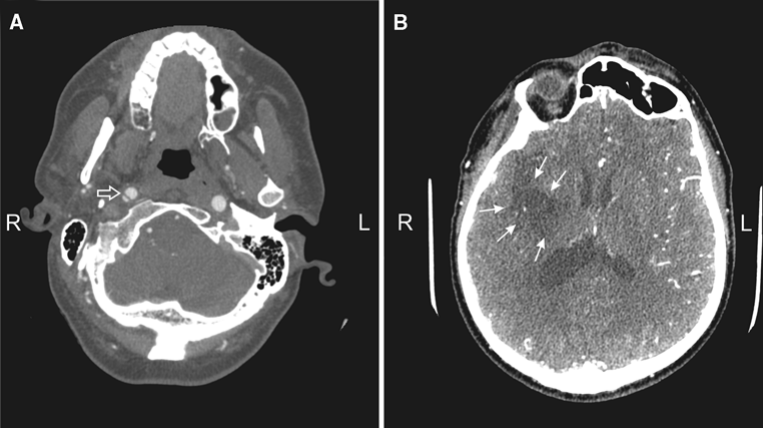
Computer tomography (CT) of the neck and head. **a** Segmental dissections of the right internal carotid artery. **b** Infraction involving deep structures of the right hemisphere including thalamus, genu and posterior limb of internal capsule, which on this single scan represent a part of the ischemic area

During CT angiography (TK–64-row MDCT scanner, LightSpeed VCT, GE, Waukesha, WI) of the neck and head, segmental dissections of the right internal carotid artery (ICA) at the level of the cranial basis were discovered (Fig. 1a). The lumen of the ICA was decreased to 4 × 2.5 mm (60 % of normal flow). Cerebral CT showed a recent infarction involving the medial part of the right temporal lobe and deep structures of the right hemisphere including the thalamus, the genu and the posterior limb of the internal capsule (Fig. 1b).

**Fig. 2:**
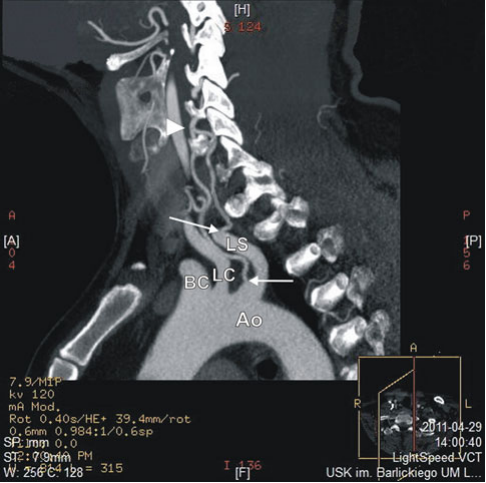
Helical CT angiography, MPR reconstruction in sagittal plane of the neck and superior part of the thorax: *arrows* origins of the duplicated left vertebral arteries, *arrowhead* level of fusion of duplicated left vertebral arteries, *Ao* aortic arch, *BC* brachiocephalic trunk, *LC* left common carotid artery, *LS* left subclavian artery

**Fig. 3:**
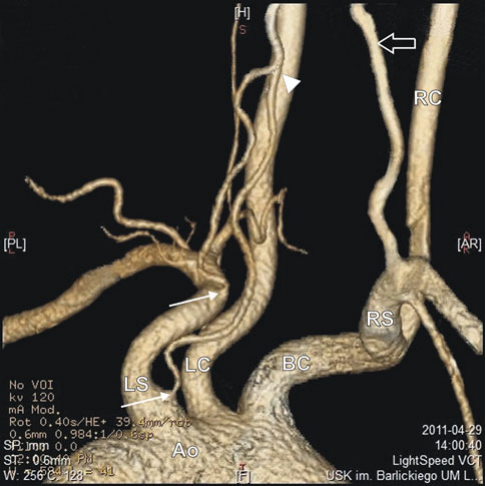
Three-dimensional CT reconstruction of the arteries. *White arrows* origins of the duplicated left vertebral arteries, *black arrow* right vertebral artery, *arrowhead* level of fusion of duplicated left vertebral arteries, *Ao* aortic arch, *BC* brachiocephalic trunk, *LC* left common carotid artery, *LS* left subclavian artery, *RC* right common carotid artery, *RS* right subclavian artery

CT angiography of the neck and head also showed a duplication of the left vertebral artery (LVA) with two origins, both from the left subclavian artery (Figs. 2, 3). The duplicated segments were fused at level of the C5–6 vertebrae into a single vertebral artery, which then entered the transverse foramen of C5 (Fig. 3). All measurements were taken on an Advantage Workstation (GE).

**Fig. 4:**
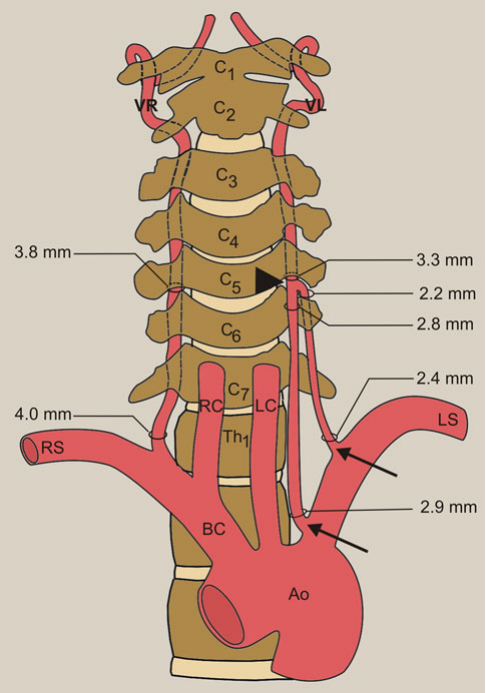
Schematic arrangements of the vessels of the neck and measurements of diameter of the vertebral arteries: *arrows* origins of the duplicated left vertebral arteries, *arrowhead* level of fusion of duplicated left vertebral arteries, *Ao* aortic arch, *BC* brachiocephalic trunk, *LC* left common carotid artery, *LS* left subclavian artery, *RC* right common carotid artery, *RS* right subclavian artery, *VL* left vertebral artery, *VR* right vertebral artery

The first LVA originated from the left subclavian artery at a distance of 7 mm from the aortic arch. It measured 2.9 and 2.8 mm in diameter at the points of origin and just before fusion, respectively (Fig. 4). The course of the artery was 101 mm. The second LVA arose from the left subclavian artery 37 mm distal to the first (44 mm from the aortic arch). It measured 2.4 and 2.2 mm in diameter at the points of origin and just before fusion, respectively (Fig. 4), and its course was 89 mm. Both vertebral arteries possessed a regular lumen. The diameter of the LVA after fusion was 3.3 mm (at level C5–6) (Fig. 4).

The right VA arose as the first branch from the left subclavian artery, 43 mm from the aortic arch. Its course was normal. The diameters of the right VA were 4 mm at the points of origin and 3.8 mm at the C5–C6 vertebrae (Fig. 4). Both thyrocervical trunks were seen to originate separately from the subclavian arteries.

**Fig. 5:**
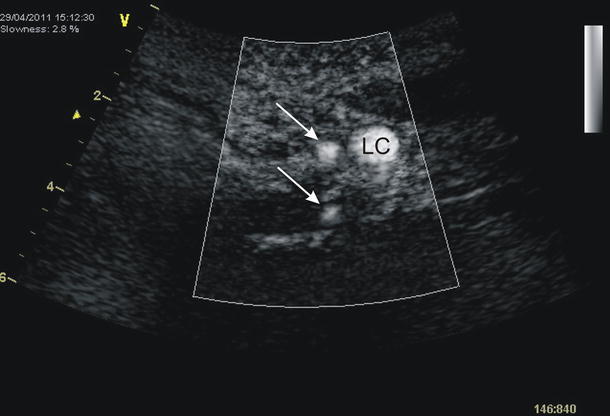
Duplication of the left vertebral artery in Doppler ultrasonography (B-flow mode): *arrows* duplicated left vertebral arteries, *LC* left common carotid artery

Additionally, complementary but independent to CT angiography Doppler Sonography B-flow mode (Vivid 7 Pro, GE) of the vertebral arteries also revealed a duplicated LVA (Fig. 5).

After all procedures were completed, Ehlers–Danlos syndrome type IV was diagnosed.

## Discussion

In most examples of a duplicated vertebral artery, the two roots originate from the aorta and the subclavian artery (Kendi and Brace 2009; Koenigsberg et al. 2003; Mahmutyazicioglu et al. 1998; Suzuki et al. 1978). However, a second, more common situation occurs when, as in the example presented in the paper, both duplicated arteries originate from the left subclavian artery (Table 1). Kiss (1968) describes a bifid origin of the right vertebral artery, one arising from the right subclavian artery and the other from the brachiocephalic trunk. Bergman et al. (1988) note the presence of dual vertebral arteries in 5 of 693 studied cadavers (0.72 %), and all were left-sided. In three of the specimens, the vertebral arteries arose as direct branches of the aortic arch, and the other two as a branch of the thyrocervical trunk. In all cases, a second ipsilateral vertebral artery arose from the subclavian artery.

**Table 1:** Reports of duplicated vertebral artery when both originate from the subclavian artery

Reference	Country	Age (years)	Sex	Side	Level of fusion	Disease and/or symptoms	Accompanying vascular anomalies
Babin and Haller (1974)	France	18	F	R	C5	Epilepsy	Dolichoarterial loop of the L vertebral artery
Hashimoto et al. (1987)	Japan	67	M	R	C5	Temporal and cerebellar infarction	None
Harada et al. (1987)	Japan	70	F	R	C4	Occipital heaviness, dizziness	Hypoplastic L vertebral artery
Takasato et al. (1992)	Japan	37	M	R	C4	Brain-stem infarction	Rudimentary and accessory L vertebral arteries
Mahmutyazicioglu et al. (1998)	Turkey	62	M	L	C2	Vertigo, weakness and nausea, thrombosis at the origin of duplicated artery	None
Goddard et al. (2001)	Great Britain	66	F	R	Mid-cervical region	Right cerebral infarct	None
Thomas et al. (2008)	United States	49	F	R	Not described	Not described	Mid-basilar trunk aneurysm
Harnier et al. (2008)	Germany	61	F	R	Not described	Dizziness	Duplication at the right common carotid artery, Fenestration of the left common carotid artery
Melki et al. (2012)	France	51	M	R	Not described	Cervical artery dissection, infarction of cerebellar vermis	None

*M* male, *F* female, *R* right, *L* left

Generally, the duplication of vertebral arteries is reported to be more common on the left side (Bergman et al. 1988; Goddard et al. 2001; Kendi and Brace 2009; Vasović 2004). However, when both arteries arise from the same subclavian artery, this variation is recognized more commonly on the right side (Table 1). Only Mahmutyazicioglu et al. (1998) and the present study describe a duplication of the LVA originating from the left subclavian artery.

Several vascular anomalies coexisting with these variations have been reported: fenestration and duplication of the common carotid artery (Harnier et al. 2008), hypoplastic vertebral artery (Harada et al. 1987), arachnoid cyst (Ionete and Omojola 2006) and aneurysm (Kendi and Brace 2009; Koenigsberg et al. 2003; Mahmutyazicioglu et al. 1998; Suzuki et al. 1978; Thomas et al. 2008).

Spontaneous dissection of the carotid or vertebral artery accounts for only about 2 % of all ischemic strokes, but 10–25 % of those occur in patients <45 years of age (Schievink 2001). Origin duplication of the VA is a relatively rare, clinically silent condition. However, some authors have speculated that fenestration and duplication of arteries is caused by “structures that split the flow and therefore divide the lumen”. Hence, it appears possible that a misplaced squamous epithelium may cause such a split in blood flow or interfere with the complete fusion of embryonic brain-stem arteries, leading to a persistent duplication (Oldendorf 1989).

Drapkin (2000) and Nogueira et al. (1997) state that duplication of vertebral artery is clinically significant because it can be mistaken for a VA dissection, and therefore has therapeutic implications for interventional procedures using the proximal VA (V1 segment of the VA). Also, Gaskill et al. (1996) consider that duplicated vertebral arteries may be more predisposed to dissection. Dare et al. (1997) also report extensive vertebrobasilar CAD on a duplicated vertebral artery.

Schievink (2001) note that well-characterized genetic hereditary affections, such as autosomal dominant connective tissue disorders (Ehlers–Danlos syndrome type IV, Marfan’s syndrome, autosomal dominant polycystic kidney disease, and osteogenesis imperfecta type I), might predispose the patient to dissections of arteries. This view is also supported by a report by Arnold et al. (2006); however, clinical signs of these disorders are present only in 1–5 % of carotid artery dissection (CAD) cases (Schievink 2001). Fibromuscular dysplasias are frequently identified in about 11 % of patients with an sVAD (Arnold et al. 2006) and in 15 % of patients with a spontaneous CAD (Schievink 2001). These segmental nonatherosclerotic noninflammatory arterial diseases of unknown etiology commonly involve the renal and carotid arteries (Schievink 2001).

In 2001, Brandt et al. stated that, as the mechanical stability and elasticity of the vessel wall is provided by connective tissue elements, structural deviations in the main components, collagen and elastic fibers, may lead to functional impairment, pre-disposing to dissection of the arterial wall at given points of minor resistance. This is supported by Brandt et al. (2005), in histopathological studies of skin biopsy samples in patients with CAD. Scientists have discovered underlying ultrastructural abnormalities similar to those seen in patients with known hereditary connective tissue diseases. They also showed that about 55 % had an underlying aberrant ultrastructural connective tissue disorder, whereas only 3 % had clinical manifestation of connective tissue disorder such as Marfan syndrome. At least 5 % of patients with CAD have a member of the family affected with CAD (Schievink et al. 1996).

The embryogenesis of the vertebral artery takes place between 32 and 40 days of development. At the 4 mm embryo stage, there are seven cervical intersegmental arteries, arising bilaterally from left and right primitive aortic arches. At the 7–12 mm stage, vascular connections between them exist. In this way the vertebral arteries are formed as fusion of the longitudinal anastomoses of the cervical intersegmental arteries. In the normal situation almost all intersegmental arteries regress, except the seventh, which forms the proximal portion of the subclavian artery, including the point of origin of the vertebral artery. Duplication of the vertebral arteries results from lack of regression of the right or left fifth intersegmental artery (Lie 1972; Padget 1948; Sim et al. 2001).

Understanding the topography and variations of the great vessels of the aortic arch is important for both the endovascular interventionist and the diagnostic radiologist. Thanks to modern imaging techniques, this knowledge has become more important in the era of carotid artery stents, vertebral artery stents, and new therapeutic options for intercranial interventions. To the best of our knowledge, extracranial duplication of the vertebral artery in patients with a coexisting spontaneous dissection of the ICA with Ehlers–Danlos syndrome has not been reported previously.

The lumen of the parts of the duplicated vessel were found to be smaller than normal. In this event, interventional procedures should be performed from the normal side if possible.
